# Incidence, Correlates, and Prognostic Significance of Mixed Response in Advanced Non-small Cell Lung Cancer

**DOI:** 10.1093/oncolo/oyad335

**Published:** 2024-01-11

**Authors:** David E Gerber, Yating Wang, Suresh S Ramalingam, Sheena Bhalla, Zhuoxin Sun, Hossein Borghaei, Julie R Brahmer, Joan H Schiller

**Affiliations:** Harold C. Simmons Comprehensive Cancer Center, UT Southwestern Medical Center, Dallas, TX, USA; Department of Internal Medicine, UT Southwestern Medical Center, Dallas, TX, USA; Peter O’Donnell Jr. School of Public Health, UT Southwestern Medical Center, Dallas, TX, USA; Eastern Cooperative Oncology Group Statistical Center, Boston, MA, USA; Dana-Farber Cancer Institute, Boston, MA, USA; Winship Cancer Institute, Emory University, Atlanta, GA, USA; Harold C. Simmons Comprehensive Cancer Center, UT Southwestern Medical Center, Dallas, TX, USA; Department of Internal Medicine, UT Southwestern Medical Center, Dallas, TX, USA; Eastern Cooperative Oncology Group Statistical Center, Boston, MA, USA; Dana-Farber Cancer Institute, Boston, MA, USA; Fox Chase Cancer Center, Philadelphia, PA, USA; University of Virginia, Charlottesville, VA, USA; University of Virginia, Charlottesville, VA, USA

**Keywords:** chemotherapy, lung cancer, mixed response, multivariate analysis, survival

## Abstract

**Background:**

Mixed response (MR), a scenario featuring discordant tumor changes, has been reported primarily with targeted therapies or immunotherapy. We determined the incidence and prognostic significance of MR in advanced non–small cell lung cancer (NSCLC) treated with cytotoxic chemotherapy.

**Patients and Methods:**

We analyzed patient-level data from ECOG-ACRIN E5508 (carboplatin-paclitaxel + bevacizumab induction followed by randomization to maintenance therapy regimens). For patients with at least 2 target lesions and available measurements after cycle 2, we characterized response as homogeneous response (HR, similar behavior of all lesions), MR (similar behavior but >30% difference in magnitude of best and least responding lesions), or true mixed response (TMR, best and least responding lesions showing different behavior: ≥10% growth versus ≥10% shrinkage). We compared category characteristics using Mann-Whitney *U* and Chi-square tests, and overall survival (OS) using log-rank test and Cox models.

**Results:**

Among 965 evaluable patients, HR occurred in 609 patients (63%), MR in 208 (22%), and TMR in 148 (15%). Median OS was 13.6 months for HR, 12.0 months for MR, and 7.6 months for TMR (*P* < .001). Compared to HR, TMR had inferior OS among stable disease cases (HR 1.62; 95% CI, 1.23-2.12; *P* < .001) and a trend toward inferior OS among progressive disease cases (HR 1.39; 95% CI, 0.83-2.33; *P* = .2). In multivariate analysis, TMR was associated with worse OS (HR 1.48; 95% CI, 1.22-1.79; *P* < .001).

**Conclusion:**

True mixed response occurs in a substantial minority of lung cancer cases treated with chemotherapy and independently confers poor prognosis.

## Teaser

Mixed response (MR), a scenario featuring discordant tumor changes, has been reported primarily with targeted therapies or immunotherapy. This article focuses on the incidence and prognostic significance of MR in advanced non–small cell lung cancer treated with cytotoxic chemotherapy.

**Implications for Practice**

This study demonstrates that mixed response (MR) occurs in a substantial minority of lung cancer cases treated with chemotherapy and independently confers poor prognosis. The clinical implications of these observations are substantial and include considerations for clinical and radiographic disease monitoring, as well as second-line therapy. These findings also raise numerous questions for future research, including molecular biologic mechanisms underlying MR as well as the optimal therapeutic approach to this clinical scenario.

## Introduction

Unusual patterns of radiological response have received increased attention in recent years. In advanced non–small cell lung cancer (NSCLC) harboring genomic driver alterations, single-site or oligo-progression may represent heterogeneous molecular evolution. In such cases, local therapy with stereotactic radiation therapy or surgical resection, with continuation of prior systemic therapy, may result in prolonged disease control.^1,2^ In advanced NSCLC and other malignancies treated with immune checkpoint inhibitors, atypical responses such as pseudoprogression occur in a meaningful minority of cases.^3-5^ This phenomenon, attributed to an early influx of antitumor immune cells, refers to the transient enlargement of tumor sites, followed by subsequent shrinkage. Compared to other systemic therapies, immune checkpoint inhibitors have also been associated with increased rates of hyperprogression, a term most commonly defined as a doubling or more of the pretreatment tumor growth rate.^6,7^

In the era of molecularly targeted therapy and immunotherapy, clinicians have also reported patterns of mixed response (MR).^8^ Some studies have identified a positive prognostic effect^9^; others, detrimental.^10,11^ Potentially confounding and limiting the interpretation of these reports, some studies describe small, single-center cohorts.^12,13^ Some include a variety of cancer therapies,^10^ while others employ atypical imaging modalities, such as serial positron emission tomography-computed tomography (PET-CT).^14^ Most obtain tumor dimensions and assign response categories retrospectively. Additionally, the MR literature includes several definitions of MR, thereby rendering cross-study comparisons difficult.

Given the growing clinical interest in but limited data informing the approach to MR in lung cancer, we analyzed heterogeneous radiographic response patterns in a large, multicenter phase III trial of chemotherapy-based treatment for advanced NSCLC.

## Methods

### Data Source and Collection

For this analysis, we analyzed patient-level data from the Eastern Cooperative Oncology Group-American College of Radiology (ECOG-ACRIN) E5508 clinical trial.^15^ This trial (NCT01107626) enrolled patients with previously untreated advanced nonsquamous NSCLC not known to harbor activating *EGFR* or *ALK* alterations with performance status ECOG 0-1. Patients received up to 4 cycles of induction carboplatin-paclitaxel + bevacizumab treatment at standard dose and schedule (carboplatin AUC 6, paclitaxel 200 mg/m^2^, bevacizumab 15 mg/kg every 3 weeks). Baseline CT scans were performed within 28 days of trial registration. CT scans were then performed every 2 cycles during this phase to assess treatment efficacy by Response Evaluation Criteria in Solid Tumors (RECIST) version 1.1. Patients tolerating treatment and experiencing clinical benefit (stable or responding disease) after 4 cycles were randomized to one of 3 maintenance arms: (A) bevacizumab, (B) pemetrexed, and (C) bevacizumab plus pemetrexed. Treatment was continued until disease progression or intolerable toxicity, with CT scans performed every 3 cycles during maintenance therapy. We selected this trial for the present study due to its large size, use of contemporary RECIST parameters, and availability of long-term follow-up data.

A total of 1516 patients were enrolled in E5508. Clinical data, including RECIST measurements, were entered into a Medidata Rave database. RECIST measurements were performed at the treating site without central input or confirmation. For the primary endpoint of overall survival (OS), there was no significant difference among the 3 maintenance treatment arms.

For each patient, we prospectively collected tumor measurements of the target non-nodal and target nodal lesions at baseline and after cycle 2. The analysis included patients who had at least 2 lesions for which measurements were available at baseline and after cycle 2. The increase or decrease for each individual lesion was calculated in percentages. We also collected demographic, tumor, treatment, and OS data.

The maximum difference in response between individual lesions was determined as a continuous variable. As described previously,^11^ based on the observed differences between the best-responding lesion and the least-responding lesion, we classified radiographic response heterogeneity on a patient level as follows: MR: >30% difference in the best-responding lesion and the least-responding lesion, with all lesions demonstrating similar behavior (either responding or progressing). True mixed response (TMR): the best-responding lesion and the least-responding lesion showing different behavior (≥10% growth versus ≥10% shrinkage). Homogeneous response (HR): similar behavior of all lesions. To arrive at these thresholds and definitions, the authors tested various cutoff values for each category. The model showing the best distribution of patients, the most significant differences in OS curves, and the largest absolute difference in median OS (mos) was deemed most discriminative. Nontarget lesions were not factored into these categories (although they were incorporated into RECIST categories).

We selected this approach—previously evaluated in patients with metastatic colorectal cancer—because it (a) can be performed using patient-level clinical trial data, (b) does not require rereview of radiographic images, (c) relies on standard radiographic assessment (eg, CT), and (d) had been developed using one of the largest cohorts (N = 290) ever evaluated for MR.^11^ By contrast, other published approaches to MR either examined only longitudinal dynamics of MR cases without comparison to other response categories,^8^ incorporated PET imaging,^10^ considered only cases with RECIST progressive disease (PD),^9^ or limited the analysis to lesions in different organs.^16^

Using RECIST version 1.1 criteria, the best overall radiographic response was categorized as complete response (CR), partial response (PR), stable disease (SD), and PD.

### Statistical Analysis

Baseline characteristics were compared among the HR, MR, and TMR groups using Mann-Whitney *U* tests for continuous data and Pearson’s Chi-square for categorical data. We calculated the incidence of HR, MR, and TMR. We estimated the median OS for each response category using the Kaplan-Meier method and used the log-rank test to compare OS curves among the 3 response groups. We performed univariate and multivariate analyses using a Cox proportional hazards model. Variables with *P* < .05 in univariate analyses were selected for multivariate analyses.

## Results

**Figure 1.**
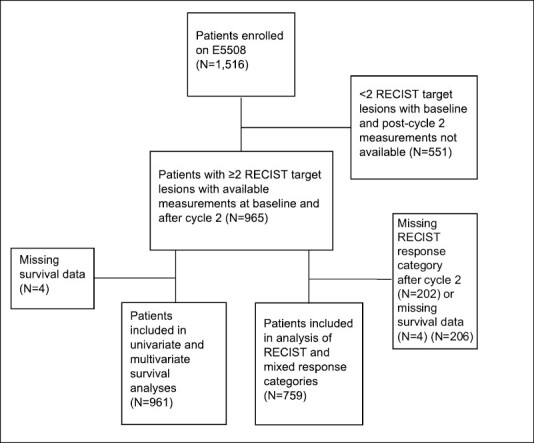
CONSORT diagram for survival and RECIST analyses.

In total, 965 patients had 2 or more RECIST target lesions with baseline and post-cycle 2 measurements and were therefore considered for analysis (Fig. 1). Mean age was 64 years, and 52% were women. Additional case characteristics, overall and according to response pattern, are shown in Table 1. HR occurred in 609 patients (63%), MR in 208 patients (22%), and TMR in 148 patients (15%). Response heterogeneity pattern was significantly associated with stage, mediastinal nodal metastases, maintenance treatment arm, and RECIST response. Specifically, patients with stage IV M1b, mediastinal nodal involvement, and PR were more likely to have MR. Patients with TMR were less likely to receive maintenance therapy than were patients with HR or MR.

**Table 1.** Baseline characteristics according to response heterogeneity category.

		Mean ± SD or number (%)				*P-*value
		HR	MR	TMR	Total	
		(*N* = 609)	(*N* = 208)	(*N* = 148)	(*N* = 965)	
Age	Mean (SD)	63.7 ± 9.4	62.9 ± 9.0	63.3 ± 8.8	63.5 ± 9.2	.47
Gender	Male	313 (51)	110 (53)	83 (56)	506 (52)	.59
	Female	296 (49)	98 (47)	65 (44)	459 (48)	
RECIST best response	CR	3 (0)	2 (1)	0 (0)	5 (1)	<.001
	PR	221 (36)	115 (55)	23 (16)	359 (37)	
	SD	182 (30)	21 (10)	63 (43)	266 (28)	
	PD	46 (8)	13 (6)	27 (18)	86 (9)	
	Unevaluable/Unknown	157 (26)	57 (27)	35 (24)	249 (26)	
ECOG	0	254 (42)	76 (37)	57 (39)	387 (40)	.39
	1	355 (58)	132 (63)	91 (61)	578 (60)	
Stage	IIIB	18 (3)	4 (2)	0 (0)	22 (2)	.04
	IV M1a	166 (27)	36 (17)	33 (22)	235 (24)	
	IV M1b	398 (65)	155 (75)	110 (74)	663 (69)	
	Recurrent	26 (4)	13 (6)	5 (3)	44 (5)	
	Unknown	1 (0)	0 (0)	0 (0)	1 (0)	
Smoking history	Current	268 (44)	101 (49)	79 (53)	448 (46)	.32
	Former	288 (47)	90 (43)	58 (39)	436 (45)	
	Never	53 (9)	17 (8)	11 (7)	81 (8)	
Maintenance therapy arm	Bevacizumab	135 (22)	45 (22)	34 (23)	214 (22)	<.001
	Pemetrexed	149 (24)	55 (26)	16 (11)	220 (23)	
	Bevacizumab + pemetrexed	136 (22)	50 (24)	26 (18)	212 (22)	
	None	189 (31)	58 (28)	72 (49)	319 (33)	
Histology	Other	60 (10)	24 (12)	15(10)	99 (10)	.77
	Adenocarcinoma	549 (90)	184 (88)	133 (90)	866 (90)	
Hilar nodal metastases	Absent	254 (42)	82 (39)	64 (43)	400 (41)	.96
	Present	342 (56)	121 (58)	81 (55)	544 (56)	
	Unknown	13 (2)	5 (2)	3 (2)	21 (2)	
Mediastinal nodal metastases	Absent	211 (35)	53 (25)	58 (39)	322 (33)	.01
	Present	390 (64)	153 (74)	85 (57)	628 (65)	
	Unknown	8 (1)	2 (1)	5 (3)	15 (2)	
Brain metastases	Absent	527 (87)	175 (84)	121 (82)	823 (85)	.61
	Present	68 (11)	26 (12)	22 (15)	116 (12)	
	Unknown	14 (2)	7 (3)	5 (3)	26 (3)	

Abbreviations: CR, complete response; ECOG, Eastern Cooperative Oncology Group; HR, homogeneous response; MR, mixed response; PR, partial response; PD, progressive disease; SD, stable disease; TMR, true mixed response.

**Figure 2.**
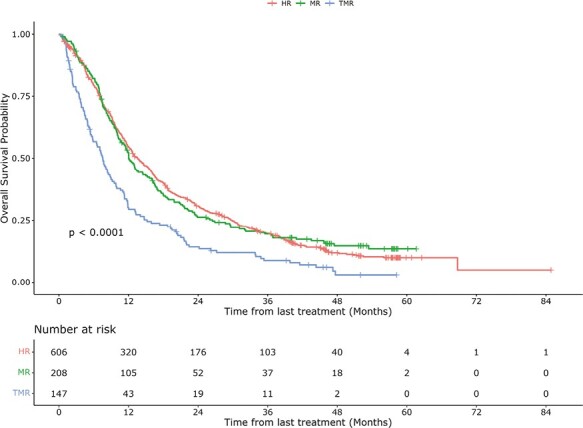
Overall survival according to response heterogeneity category. Abbreviations: HR, homogeneous response; MR, mixed response; TMR, true mixed response.

Overall survival according to response heterogeneity category is shown in Fig. 2. Four patients with missing survival time data were excluded from the analysis. Median OS for the entire cohort was 12.0 (95% CI, 11.3-13.0) months. By category, median OS was 13.6 months (95% CI, 12.3-15.5 months) for HR, 12.0 months (95% CI, 10.7-15.2 months) for MR, and 7.6 months (95% CI, 5.8-9.2 months) for TMR.

**Figure 3.**
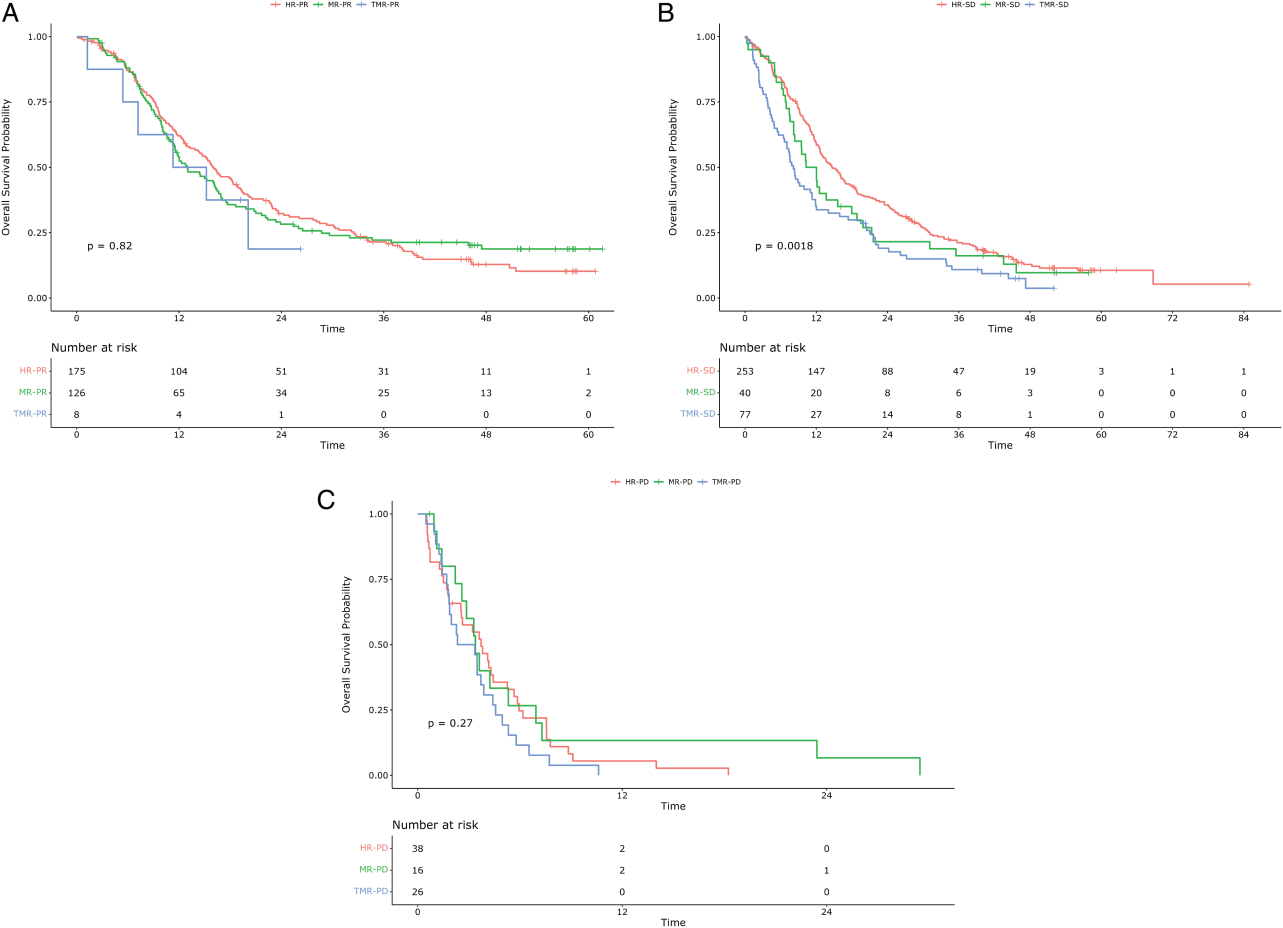
Overall survival according to response heterogeneity and RECIST response categories. (**a**) RECIST PR, (**b**) RECIST SD, (**c**) RECIST PD. Abbreviations: HR, homogeneous response; MR, mixed response; PD, progressive disease; PR, partial response; RECIST, Response Evaluation Criteria in Solid Tumors; SD, stable disease; TMR, true mixed response.

Overall survival according to both response heterogeneity and RECIST response is shown in Table 2 and Fig. 3. This analysis included 759 patients with available response heterogeneity, post-cycle 2 RECIST response, and OS data. We observed certain differences in RECIST response according to response heterogeneity. For instance, RECIST PD was more common in patients with TMR (23%) than among patients with MR (9%) or HR (8%). Conversely, RECIST PR occurred more frequently among patients with MR (69%) than in patients with HR (38%) and TMR (7%). Within each RECIST response category (apart from CR, of which there were only 2 cases), median OS was numerically worse for MR and TMR cases than for HR cases. In the RECIST SD category, survival was significantly shorter for TMR than for HR cases: HR 1.62 (95% CI, 1.23-2.12; *P* < .001). In the RECIST PD category, this difference did not reach statistical significance: HR 1.39 (95% CI, 0.83-2.33; *P* = .2).

**Table 2.** Overall survival according to response heterogeneity and RECIST response category.

Response heterogeneity	RECIST response	*N*	Median OS (95% CI)
HR	PR	175	16.1 (13.8-19.2)
	SD	253	14.7 (12.7-17.6)
	PD	38	3.7 (2.5-5.7)
MR	PR	126	12.9 (11.5-16.7)
	SD	40	11.2 (8.2-18.9)
	PD	16	3.4 (2.6-7.3)
TMR	PR	8	13.3 (7.2-NA)
	SD	77	8.2 (6.6-11.9)
	PD	26	2.8 (1.8-4.6)

Abbreviations: CI, confidence interval; CR, complete response; HR, homogeneous response; MR, mixed response; OS, overall survival; PR, partial response; PD, progressive disease; SD, stable disease; TMR, true mixed response.

In both univariate and multivariate analysis, male gender, M1b staging, ECOG 1 (versus 0), nonadenocarcinoma histology, mediastinal nodal metastasis, no administration of maintenance therapy, and TMR were significantly associated with worse OS (Table 3). There was no significant difference in OS between HR and MR.

**Table 3.** Univariate and multivariate analyses for OS.

Characteristic	Univariate analysis		Multivariate analysis	
	HR (95% CI)	*P-*value	HR (95% CI)	*P*-value
Gender				
Male	Reference		Reference	
Female	0.74 (0.65-0.85)	<.001	0.78 (0.68-0.90)	.001
Smoking history				
Current	Reference			
Former	0.90 (0.78-1.03)	.13		
Never	0.79 (0.61-1.02)	.08		
Stage				
IIIB	Reference		Reference	
IV M1a	1.44 (0.85-2.44)	.18	1.70 (1.00-2.90)	.05
IV M1b	2.21 (1.32-3.70)	.003	2.34 (1.39-3.94)	.001
Recurrent	1.69 (0.92-3.09)	.09	1.62 (0.88-2.98)	.12
Unknown	7.48 (0.98-56.91)	.05	7.20 (0.91-57.31)	.06
ECOG				
0	Reference		Reference	
1	1.45 (1.3-1.7)	<.001	1.38 (1.20-1.59)	<.001
Histology				
Other	Reference		Reference	
Adenocarcinoma	0.738 (0.59-0.92)	.007	0.74 (0.59-0.93)	.009
Hilar nodal metastases				
Absent	Reference			
Present	1.09 (0.97-1.2)	.17		
Mediastinal nodal metastases				
Absent	Reference		Reference	
Present	1.14 (1-1.3)	.04	1.15 (1.00-1.31)	.05
Brain metastases				
Absent	Reference			
Present	1.04 (0.88-1.2)	.66		
Maintenance therapy arm				
Bevacizumab	Reference		Reference	
Pemetrexed	0.87 (0.71-1.07)	.17	0.85 (0.69-1.04)	.12
Bevacizumab + pemetrexed	0.89 (0.72-1.09)	.26	0.89 (0.73-1.10)	.30
None	2.36 (1.96-2.84)	<.001	2.30 (1.90-2.77)	<.001
Response heterogeneity category				
HR	Reference		Reference	
MR	0.99 (0.83-1.18)	.91	0.91 (0.76-1.09)	.3
TMR	1.71 (1.41-2.06)	<.001	1.48 (1.22-1.79)	<.001

We also specifically examined cases featuring the emergence of new nontarget lesions. Although not considered in MR definitions, this scenario is considered disease progression by RECIST. Overall, 76 cases (8%) had new lesions documented. Among these, 41 cases (54%) were HR, 13 cases (17%) were MR, and 22 cases (29%) were TMR—approximately twice the frequency of TMR cases in the overall study population (15%).

## Discussion

Given the heightened awareness of and attention to MR in patients treated with targeted therapies and immune checkpoint inhibitors, it is critical to place this clinical and radiographic phenomenon in context. We therefore conducted, to our knowledge, the largest study to date of MR in lung cancer or any malignancy. In the present analysis, we analyzed almost 1000 patients with advanced nonsquamous NSCLC treated initially with carboplatin-paclitaxel plus bevacizumab therapy. We observed that MR (target lesions with similar behavior but > 30% difference in individual lesion responses) occurred in over 20 percent of cases. True mixed response (ie, different behavior [growth and shrinkage] of target lesions) occurred in 15% of patients overall and in almost 30% of cases featuring the appearance of new (nontarget) lesions. In the overall population study population, these individuals had inferior OS. Significantly worse outcomes were also seen among the subset of cases with RECIST SD, with a clear but nonsignificant trend noted for the subset of cases with RECIST disease progression. In multivariate analysis controlling for numerous patient and tumor characteristics, TMR was associated with a nearly 50% increase in the rate of death compared to HR and MR cases.

The incidence of MR and TMR in this chemotherapy-treated population is noteworthy. Mixed or dissociated responses have largely been considered phenomena restricted to patients treated either with immune checkpoint inhibitors or with molecularly targeted therapies. Indeed, the 15% rate of TMR in the present study is approximately twice the rate of dissociated response observed in patients with NSCLC receiving single-agent anti-PD1/PDL1 therapy (8%).^5^ We recognize the limitations of comparing our current findings with studies using different definitions of response heterogeneity, as the case for this immune checkpoint inhibitor-treated population. Nevertheless, one might surmise that the actual difference is even greater. The “dissociated response” used in the immunotherapy study requires only a different directionality of tumor behavior (growth vs. shrinkage). However, the “true mixed response” used in the present analysis requires a minimum change (≥10%) in each direction. Notably, our reported rate of TMR also numerically exceeds that in a chemotherapy-treated colorectal cancer population using the same parameter (9%).^11^

The underlying mechanism of MRs in the current study of cytotoxic therapy remains unclear. Previously reported series in lung cancer have identified discordant molecular evolution of classical *EGFR* mutations (eg, *EGFR* T790M, wild-type *EGFR,* and *MET* alterations) during the process of metastasis and among cases with MRs,^10,17^ but such genomic considerations seem unlikely to apply to the present study’s population of *EGFR* wild-type NSCLC treated with cytotoxic and antiangiogenic agents. Mixed responses to immunotherapy have been attributed to tissue-dependent tumor microenvironments.^18^ Among surgically resected localized lung adenocarcinomas, those with greater intratumoral heterogeneity (assessed by multiregion whole exome sequencing) were more likely to recur,^19^ consistent with a worse prognosis independent of medical therapy. However, the association between intratumoral heterogeneity—a metric based on a single tumor—and MR among 2 or more lesions is not known.

The adverse prognostic impact of MR, particularly TMR, is striking. In a study of NSCLC cases treated with various targeted and cytotoxic therapies, MR characterized by PET was associated with worse outcomes.^10^ What is particularly striking about our current findings is that MR, specifically TMR, tended to have inferior survival across RECIST response categories. One might expect this observation among cases with RECIST PR; an enlarging lesion among others that are clearly shrinking or unchanged might indicate near-term emergence of broader therapeutic resistance. But the worse outcomes of TMR cases within the RECIST SD and PD categories is a more complex consideration. For instance, why a progressing cancer with some shrinking lesions would have inferior survival to a progressing cancer featuring uniformly enlarging lesions is not known. One potential explanation is that, due to biologic heterogeneity, cancers demonstrating MR are difficult to treat with any medical therapy, including subsequent lines of treatment. This could be particularly true when the initial MR occurs in the setting of a multiagent regimen—in this study, a platinum, taxane, and antiangiogenic agent. Nonmedical, local treatment modalities such as radiation therapy or surgical resection, either in the setting of oligoprogression or persistence at the time of expected best response,^20-23^ may help address these challenges.

Strengths of this study include the large sample size, the prospective collection of RECIST tumor dimensions, the uniform nature and timing of serial imaging studies, the duration of follow-up, and the availability of detailed clinical data such as sites of metastases. Main weaknesses include lack of tumor genomic characterization (beyond the absence of activating *EGFR* and *ALK* alterations), inclusion of only cytotoxic and antiangiogenic therapies, lack of information on postprogression therapy, and exclusion of squamous tumors. Although patients in the E5508 trial we analyzed were assigned to one of 3 maintenance therapy regimens, there was no difference in survival among these treatments; accordingly, maintenance therapy assignment seems unlikely to influence our findings. Because our classification of response heterogeneity incorporated only target lesion data, it is also possible that we underestimated the true incidence of MR, as some cases might have shrinking target lesions concurrent with the appearance of new or unequivocal growth of nontarget lesions. Finally, we recognize that the present analysis only applies to cases with multiple RECIST target lesions, a population that might be expected to have worse outcomes than cases with only a single lesion based on the association between radiographic tumor burden and survival in advanced NSCLC.^24^

We also acknowledge that our approach to define MR, while based on previously published methodology,^11^ may not represent the optimal characterization of this phenomenon. For instance, in cases of very small target lesions, differences of 10% in longest diameters would be expected to have relatively large margins of error—a shortcoming that might be addressed by mandating minimum absolute changes in addition to proportional changes, as required by RECIST. It is also possible that different category definitions (eg, discordant changes other than ±10%, or differences in concordant changes other than 30%) may have had greater discriminative capacity in this study population. The focus of the present study, however, was not the development of a new metric. Instead, we sought to apply a previously established method to a new clinical scenario.

## Conclusion

Although divergent radiographic responses have gained attention primarily among tumors treated with molecularly targeted therapies or immune checkpoint inhibitors, they also occur in a substantial proportion of cancers treated with conventional, cytotoxic chemotherapy. True mixed response—a scenario in which some tumor lesions shrink while others grow—is independently associated with poor prognosis. Within RECIST categories, it has worse outcomes than HRs. These findings highlight the clinical importance of divergent radiographic response patterns across treatment types. Further research into the biologic underpinnings and optimal treatment of MR is warranted.

## Data Availability

The data underlying this article were provided by ECOG-ACRIN under licence/by permission. Data will be shared on request to the corresponding author with permission of ECOG-ACRIN.
